# Temperature Drift Compensation for Hemispherical Resonator Gyro Based on Natural Frequency

**DOI:** 10.3390/s120506434

**Published:** 2012-05-15

**Authors:** Xu Wang, Wenqi Wu, Zhen Fang, Bing Luo, Yun Li, Qingan Jiang

**Affiliations:** 1 College of Mechanical Engineering and Automation, National University of Defense Technology, Changsha 410073, Hunan Province, China; E-Mails: wenqiwu_lit@hotmail.com (W.W.); ruobing@nudt.edu.cn (B.L.); ly20090801@163.com (Y.L.); jqa1987@163.com(Q.J.); 2 Institution of Piezoelectric and Acousto-optic Technology, Chongqing 400060, China; E-Mail: hrg@sipat.com

**Keywords:** Hemispherical Resonator Gyro (HRG), natural frequency, temperature compensation, drift

## Abstract

Temperature changes have a strong effect on Hemispherical Resonator Gyro (HRG) output; therefore, it is of vital importance to observe their influence and then make necessary compensations. In this paper, a temperature compensation model for HRG based on the natural frequency of the resonator is established and then temperature drift compensations are accomplished. To begin with, a math model of the relationship between the temperature and the natural frequency of HRG is set up. Then, the math model is written into a Taylor expansion expression and the expansion coefficients are calibrated through temperature experiments. The experimental results show that the frequency changes correspond to temperature changes and each temperature only corresponds to one natural frequency, so the output of HRG can be compensated through the natural frequency of the resonator instead of the temperature itself. As a result, compensations are made for the output drift of HRG based on natural frequency through a stepwise linear regression method. The compensation results show that temperature-frequency method is valid and suitable for the gyroscope drift compensation, which would ensure HRG's application in a larger temperature range in the future.

## 1. Introduction

The hemispherical resonator gyro (HRG) is a solid state gyroscope whose sensing property is based on a standing vibration wave precession. It has the features of high accuracy, long life span, inherent high reliability, natural radiation hardness and no parts that can wear out. With its excellent performance, the Scalable Space Inertial Reference Unit (Scalable SIRU) and its predecessor, the Space Inertial Reference Unit (SIRU), which all are made from HRGs, have been launched on more than 125 spacecraft missions for NASA, Department of Defense, commercial and international space applications [1,2]. It is reported that HRGs have already achieved 18 million h of continuous operation with 100 percent mission success in various space and military application tasks.

The HRG contains three primary functional components: the hemispherical resonator, the forcer and the pickoff. They are all made of quartz and bonded together within a sealed vacuum housing [3,4].

The temperatures of both the inner house and resonator will change due to the heat produced through the vibration of the resonator and ambient temperature changes of the HRG. Moreover, owing to the uneven heat conduction, a temperature gradient will exist in the vacuum housing of the HRG. Since factors such as temperature changes and temperature gradient can strongly result in temperature drifts which seriously affect HRG's application in commercial and military areas, it is of great importance to suppress or compensate these temperature drifts. At present, there are mainly two methods to suppress the drift caused by temperature changes [5]:

1. Temperature stabilization method: the HRG is placed in a controlled temperature chamber, that can keep the surrounding temperature constant and provide the best conditions for the gyro, which decreases the drift resulting from temperature changes.
2. Temperature compensation method based on a math drift model of the HRG: obtain a curve about the relationship between the output of the HRG and temperature and make compensations on-board through software.

As for the first method, the sensing components (resonator) of the gyro are encapsulated in a vacuum, so basic modes of heat exchange could only depend on the thermal radiation and the heat transfer through the sustaining pole between the resonator and the outside cover, causing the temperature of sensing components to change slowly. As a result, it takes a long time to make the gyro sensing components' temperature approach the pre-set temperature of the controlled temperature chamber before it could work, so it could not meet the needs of rapid reaction. Furthermore, the temperature control system will greatly increase the volume, weight and cost which would make the strapdown inertial navigation system much too expensive. One point worth mentioning is that volume and weight are two decisive factors in space applications, so big volume and weight are regarded as fatal limitations.

Compared with the first method, the latter one (temperature compensation method) is much easier to adopt since it doesn't require an increase in volume, weight or hardware cost. However, the resonator is sealed in a vacuum house and any accessories attached to it for temperature sensing would seriously deteriorate its performance, making it unrealistic to set up any temperature sensor on the resonator. Although the temperature sensor could be fixed on the inner vacuum housing, the heat exchange is very slow without air. Thus, the temperature sensor attached to the inner housing is not able to represent the real time temperature of the resonator. In a word, it would be very difficult to directly measure the temperature of the resonator.

Fortunately, as references [6–8] mention, the resonator oscillation frequency of the HRG has a temperature sensitivity of about 80 ppm/°C due to the temperature coefficient of the Young's modulus of fused quartz. Since the reference phase generator of the primary control loop is locked to the resonator in the HRG, its frequency provides a direct measure of the temperature of the resonator and can be used for thermal modeling. In reference [9], Loper and Lynch point out that the resonator frequency variation is an excellent measure of its temperature variation, and they give a direct linear formula of the relationship between the resonator natural frequency and the resonator temperature, but no detailed analyses of the relationship between temperature and frequency of HRG are available. Furthermore, little attention has been focused on the gyroscope temperature compensation by using the natural frequency in the literature.

The resonator itself could serve as a high precision temperature sensor for temperature compensation of the gyroscope. In reference [10], a smart temperature sensor which employs the change of the quartz natural frequency realizes the temperature measurement with a precision of 0.01 °C. Thus, it is feasible to employ the natural frequency change of the HRG resonator to realize the temperature measurement. This method can not only improve the performance of the HRG over the whole temperature range, but is also inexpensive and easy to adopt since it needs no additional hardware.

This paper provides detailed descriptions of the relationship between the temperature and frequency of the HRG. As long as the frequency of resonator is obtained by the digital control loops of the HRG, temperature compensation for the output of the gyroscope can be realized in real time [11,12].

## 2. The Temperature-Frequency Characteristic of the HRG Resonator

References such as [13,14] describe the relationship between resonant frequency of HRG and its material parameters, but none of the references give the detailed deviation process and there are even some errors in the results of reference [13]. Those errors are corrected in this paper.

**Figure 1.**
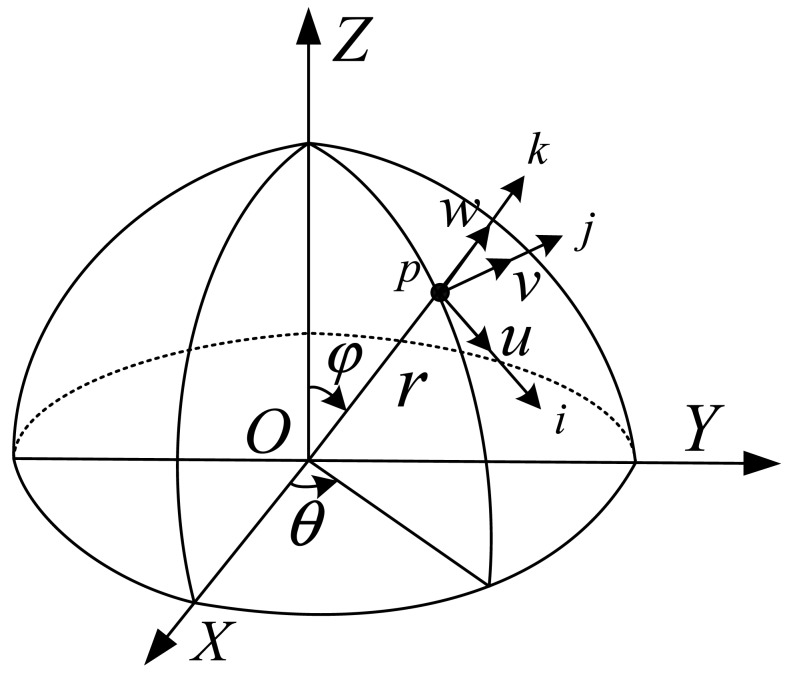
The illustration of the mean shell of the hemispherical resonator.

The energy method can be applied to determine the natural frequency of the HRG resonator, and then the temperature coefficient of the natural frequency of HRG can be obtained. A model for a thin axis-symmetrical hemispherical shell with mean radius *r* and radial thickness *h* is shown in Figure 1, which is assumed to be isotropic with free boundary conditions on the open end. When the hemisphere shell doesn't rotate, the hemispherical shell has displacements of the form [13]:

(1) $$\{\begin{array}{c} u(\varphi ,\theta ,t)=u(\varphi )\cos n\theta \cos\omega_{n}t\\ v(\varphi ,\theta ,t)=v(\varphi )\sin n\theta \cos\omega_{n}t\\ w(\varphi ,\theta ,t)=w(\varphi )\cos n\theta \cos\omega_{n}t \end{array}$$

where *u*(*φ*) is the generatrix direction displacement; *v*(*φ*) is the ring direction displacement and *w*(*φ*) is the radial direction displacement. The kinetic energy of the shell can be expressed as [15]:

(2) $$K=\frac{1}{2}r^{2}\rho h\int_{0}^{\pi /2}\int_{0}^{2\pi }({\dot{u}}^{2}+{\dot{v}}^{2}+{\dot{w}}^{2})\sin\varphi d\theta d\varphi$$

where K and respectively refer to kinetic energy and density of the shell. In addition, from the theory of elasticity, the well-known expression for the strain energy stored in a body during elastic deformation is [16,17]:

(3) $$U=\frac{1}{2}\int_{V}(\sigma_{\varphi }e_{\varphi }+\sigma_{\theta }e_{\theta }+\sigma_{n}e_{n}+\sigma_{\varphi \theta }\gamma_{\varphi \theta }+\sigma_{\varphi n}\gamma_{\varphi n}+\sigma_{\theta n}\gamma_{\theta n})dV$$

where *dV* is the element volume; *e_i_* and *γ_i_* are respectively normal strains and shear strains; *σ_i_* is the normal stresses. By applying the Kirchhoff hypothesis of thin shells, Equation (3) is reduced to:

(4) $$U=\frac{1}{2}\int_{V}(\sigma_{\varphi }e_{\varphi }+\sigma_{\theta }e_{\theta }+\sigma_{\varphi \theta }\gamma_{\varphi \theta })dV$$

When the materials are isotropic, Hooke's law is written as follows:

(5) $$\{\begin{array}{l} \sigma_{\varphi }=\frac{E}{1-\mu^{2}}(e_{\varphi }+\mu e_{\theta })\\ \sigma_{\theta }=\frac{E}{1-\mu^{2}}(e_{\theta }+\mu e_{\varphi })\\ \sigma_{\varphi \theta }=\frac{E}{2(1+\mu )}\gamma_{\varphi \theta } \end{array}$$

where E is the Young's modulus and *μ* is the Poisson ratio. The total strains at an arbitrary point in the shell can be represented as:

(6) $$\{\begin{array}{l} e_{\varphi }={ɛ}_{\varphi }+z\kappa_{\varphi }\\ e_{\theta }={ɛ}_{\theta }+z\kappa_{\theta }\\ \gamma_{\varphi \theta }={ɛ}_{\varphi \theta }+2z\tau \end{array}$$

where *ε_φ_*, *ε_θ_* and *ε_φθ_* are the normal and shear strains in the middle surface; *κ_φ_* and *κ_θ_* are the middle surface changes in curvature; *τ* is the middle surface twist and *z* measures the distance of the arbitrary point from the corresponding point on the middle surface along *k* and varies over the thickness. Then, substituting Equations (5) and (6) into Equation (4), integrating over the thickness, yields:

(7) $$U=\frac{Eh}{2(1-\mu^{2})}\int_{0}^{2\pi }\int_{0}^{\pi /2}[({ɛ}_{\varphi }^{2}+{ɛ}_{\theta }^{2}+2\mu {ɛ}_{\varphi }{ɛ}_{\theta }+\frac{1-\mu }{2}{ɛ}_{\varphi \theta }^{2}]+\frac{h^{2}}{12}(\kappa_{\varphi }^{2}+\kappa_{\theta }^{2}+2\mu \kappa_{\varphi }\kappa_{\theta }+2(1-\mu )\tau^{2})]r^{2}\sin\varphi d\varphi d\theta$$

For the case of a hemispherical shell, the middle surface strain and curvature changes in Equation (7) are:

(8) $$\{\begin{array}{ll} {ɛ}_{\varphi }=\frac{1}{r}(w+\frac{\partial u}{\partial \varphi }),\;{ɛ}_{\theta }=\frac{1}{r}(\frac{\partial v}{\partial \theta }\frac{1}{\sin\varphi }+u\cdot \text{ctg}\varphi +w),\;{ɛ}_{\varphi \theta }=\frac{1}{r}(\frac{1}{\sin\varphi }\frac{\partial u}{\partial \theta }+\frac{\partial v}{\partial \varphi }-v\cdot \text{ctg}\varphi )\\ \kappa_{\varphi }=\frac{1}{r^{2}}(\frac{\partial u}{\partial \varphi }-\frac{\partial^{2}w}{\partial \varphi^{2}}),\;\kappa_{\theta }=\frac{1}{r^{2}\sin\varphi }(-\frac{\partial^{2}w}{\partial \theta^{2}}\frac{1}{\sin\varphi }+\frac{\partial v}{\partial \theta }-\frac{\partial w}{\partial \varphi }\cos\varphi +u\cos\varphi )\\ \tau =\frac{1}{r^{2}\sin\varphi }(-\frac{\partial^{2}w}{\partial \varphi \partial \theta }+\frac{\partial w}{\partial \varphi }\text{ctg}\varphi +\frac{\partial u}{\partial \theta }+\frac{\partial v}{\partial \varphi }\sin\varphi -v\cos\varphi ) & \end{array}$$

As for free vibration of clamped-free hemispherical shell, under the condition of paucity displacement, the Lord Rayleigh inextentional condition is satisfied, so the normal stress and shear stress will be approximately reduced into zero, which is:

(9) $${ɛ}_{\varphi }={ɛ}_{\theta }={ɛ}_{\varphi \theta }=0$$

Substituting Equations (8) into Equation (9) yields [13]:

(10) $$\{\begin{array}{c} u(\varphi )=C\sin\varphi tg^{n}\frac{\varphi }{2}\\ v(\varphi )=C\sin\varphi tg^{n}\frac{\varphi }{2}\\ w(\varphi )=-C(n+\cos\varphi )tg^{n}\frac{\varphi }{2} \end{array}$$

where *C* is an arbitrary constant.

Substituting Equations (10) into Equation (1) and then the Equation (1) can be expressed as:

(11) $$\{\begin{array}{l} u=C\sin\varphi tg^{n}\frac{\varphi }{2}\cos n\theta \sin\omega_{n}t\\ v=C\sin\varphi tg^{n}\frac{\varphi }{2}\sin n\theta \sin\omega_{n}t\\ w=-C(n+\cos\varphi )tg^{n}\frac{\varphi }{2}\cos n\theta \sin\omega_{n}t \end{array}$$

Substituting Equation (11) into Equations (2) and (7), the energies stored in the shell can be obtained. Thus, expressions of the maximum kinetic and potential energy are [18]:

(12) $$\begin{array}{c} K_{\max}=\frac{1}{2}\omega_{n}^{2}\pi C^{2}r^{2}\rho h\int_{0}^{\pi /2}\{{\left(n+\cos\varphi \right)}^{2}+2\sin^{2}\varphi \}\sin\varphi tg^{2n}\frac{\varphi }{2}d\varphi \\ U_{\max}=\frac{\pi C^{2}h^{3}E}{6(1+\mu )r^{2}}n^{2}{\left(n^{2}-1\right)}^{2}\int_{0}^{\pi /2}\sin^{-3}\varphi tg^{2n}\frac{\varphi }{2}d\varphi \end{array}$$

Utilizing the condition *K*_max_ = *U*_max_, the natural frequency of the hemispherical shell can be determined, which is [13,14]:

(13) $$\omega_{n}=\frac{n(n^{2}-1)h}{r^{2}}{\left(\frac{E}{3(1+\mu )\rho }\frac{\int_{0}^{\pi /2}\sin^{-3}\varphi tg^{2n}\frac{\varphi }{2}d\varphi }{\int_{0}^{\pi /2}\{{\left(n+\cos\varphi \right)}^{2}+2\sin^{2}\varphi \}\sin\varphi tg^{2n}\frac{\varphi }{2}d\varphi }\right)}^{1/2}$$

Attention is paid to the *n* = 2 vibration mode of the resonator, and then the natural frequency can be rewritten as:

(14) $$f=\omega^{2}=\frac{2h}{r^{2}}{\left(\frac{3E}{(1+\mu )\rho }\frac{I}{J}\right)}^{1/2}$$

where the terms I and J are defined as follows:

$$\begin{array}{cc} I=\int_{0}^{\pi /2}\sin^{-3}\varphi tg^{2n}\frac{\varphi }{2}d\varphi & J=\int_{0}^{\pi /2}\{{\left(2+\cos\varphi \right)}^{2}+2\sin^{2}\varphi \}\sin\varphi tg^{2n}\frac{\varphi }{2}d\varphi \end{array}$$

I and J are only relative to the shape of the hemispherical shell and *f* denotes the natural frequency of the resonator at a certain temperature. Considering the temperature effect on the natural frequency, the Equation (14) can be rewritten as follows:

(15) $$f(T)=\frac{2h(T)}{r^{2}(T)}{\left(\frac{3E(T)}{\left(1+\mu (T))\rho (T\right)}\frac{I}{J}\right)}^{1/2}$$

From Equation (15), we can conclude that the natural frequency of the resonator is not only relative to the Young's modulus *E* but also related to the resonator's density *ρ*, Poisson ratio *μ*, thickness *h* and radius *r*. All the parameters are easily affected by the temperature, but the Young's modulus contributes most to the natural frequency changes since it is susceptible to the temperature [7]. Since a resonator's material properties are affected by temperature changes, its natural frequency would change as temperature changes. Based on the relationship between the temperature and natural frequency, the inner temperature of the HRG can be obtained through its digital frequency outputs; therefore, the temperature drift can be compensated from the natural frequency other than temperature which is hard to measure by using sensors.

However, if all the terms which are affected by temperature are respectively taken into consideration, the relationship between temperature and natural frequency will be very difficult to obtain. Therefore, in this paper, a Taylor expansion method is employed to analyze the temperature coefficient of frequency of HRG for simplicity. The frequency temperature function *f*(*T*) at the reference temperature *T*_0_ can be described as a Taylor series which is:

(16) $${f(T)=f(T_{0})+\frac{\partial f}{\partial T}|}_{T=T_{0}}{(T-T_{0})+\frac{\partial^{2}f}{2!\partial T^{2}}|}_{T=T_{0}}{{\left(T-T_{0}\right)}^{2}+\ldots \frac{\partial^{2}f}{n!\partial T^{n}}|}_{T=T_{0}}{\left(T-T_{0}\right)}^{n}+\ldots$$

Based on the theory of thermodynamics of materials, the natural frequency of quartz can be expressed as a three-order polynomial, so the high-order terms can be neglected:

(17) $${f(T)=f(T_{0})+\frac{\partial f}{\partial T}|}_{T=T_{0}}{(T-T_{0})+\frac{\partial^{2}f}{2!\partial T^{2}}|}_{T=T_{0}}{{\left(T-T_{0}\right)}^{2}+\ldots \frac{\partial^{3}f}{3!\partial T^{3}}|}_{T=T_{0}}{\left(T-T_{0}\right)}^{3}$$

Comparing Equation (15) with Equation (17), it is found that it is difficult to obtain the coefficients by analytical methods, so we obtain the coefficients by temperature experiments.

## 3. Temperature-Frequency Coefficient Calibration Experiments

Under the FTR mode of the HRG, four control loops, which are reference-phase loop, amplitude-control loop, quadrature-control loop and rebalance control loop, are employed to ensure that the HRG works at a high performance status. Reference-phase loop and amplitude-control loop are employed to maintain the primary vibration pattern at its natural frequency and at constant amplitude. The quadrature control loop which changes the stiffness of the resonator is employed to eliminate the frequency split of the two vibration modes. Simultaneously, the rebalance loop is employed to nullify the response of the second mode, and the rotation rate can be obtained from the force which is used to nullify the response of the second mode. The information including rotation rate and the vibrating frequency of the resonator are all obtained in digital form. Since the reference phase loop is locked to the resonator, its frequency change traces the temperature changes of the resonator.

**Figure 2.**
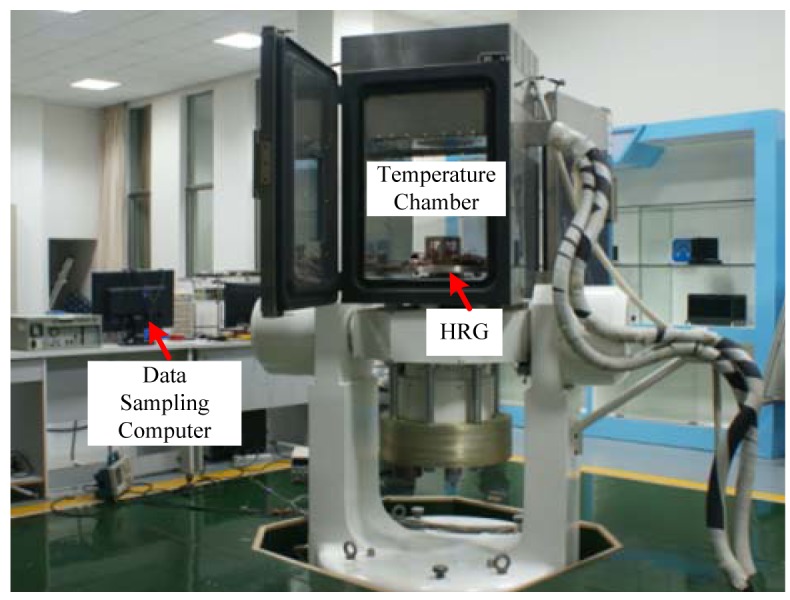
The temperature experiments carried out by a controlled temperature chamber.

Experiments are designed to get the temperature-frequency coefficient of the HRG, as shown in Figure 2. In order to obtain the temperature-frequency coefficient of the gyroscope, it was placed in a temperature chamber for 4–5 h while the the temperature ranged from −20 °C to 40 °C, respectively, in 10 °C steps. Due to the slow heat exchange of the inner gyroscope, it took a long time to make the resonator temperature identical with the pre-set ones. During this process, we recorded the frequency until it did not change any more, which denoted that the temperature of the resonator was identical with the pre-set one of the temperature chamber.

**Figure 3.**
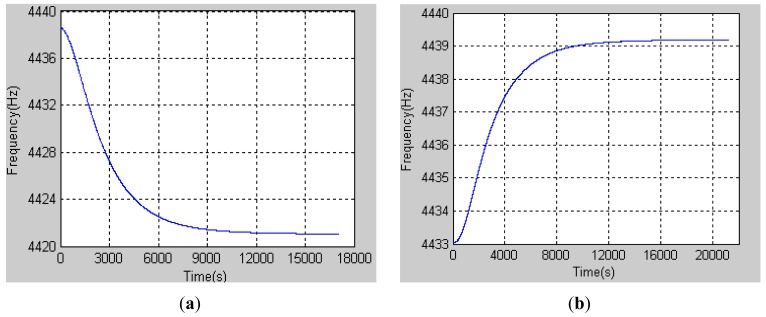
HRG natural frequency varies with temperature changes. (**a**) Frequency changes from room temperature to −10 °C; and (**b**) Frequency changes from room temperature to 40 °C.

The frequency change of the gyroscope which was placed in the temperature chamber with the temperature setting −10 °C is shown in Figure 3(a). The date sample frequency was 200 Hz with a FPGA and the duration time was nearly 5 h. As shown in the Figure 3(a), at first, the frequency decreased rapidly, but after 9,000 s, the frequency decreased slowly, which meant that the temperature was nearly stable. After 15,000 s, the frequency hardly change anymore which denoted that the resonator's temperature had reached the set temperature. Frequency of the resonator at this moment is in correspondence with the temperature −10 °C.

Similarly, Figure 3(b) shows the frequency changes when the temperature is increasing. The temperature chamber was set to 40 °C in advance and the frequency of the resonator was increasing as its temperature increased. After a long time, the frequency was stable, which meant that the heat balance in the gyroscope was established. The frequency at that moment can reasonably stand for the temperature 40 °C. As mentioned above, every temperature point repeats the similar experiment from which we can get different frequencies relative to different temperatures, which are listed in Table 1.

The coefficient of Equation (17) can be obtained through the data in Table 1 by using the least-square fitting method, which is:

(18) $$f=1.528\times 10^{-5}T^{3}-9.702\times 10^{-4}T^{2}+0.3716T+4424.901$$

**Figure 4.**
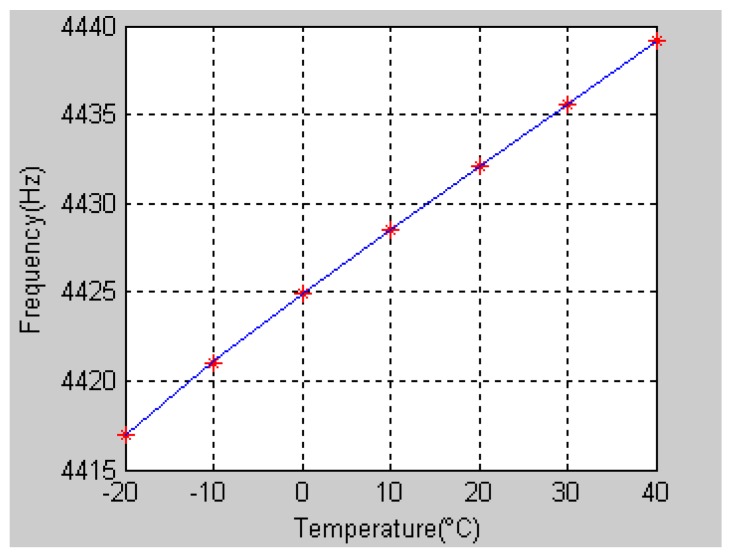
Temperature-frequency fitting curve.

The second order coefficient is 9.702 × 10^−4^ and the third order coefficient is 1.528 × 10^−5^, which means that the high order terms have little effect on the frequency. The fitting curve is shown in Figure 4, with the maximum fitting error being 0.12%.

**Figure 5.**
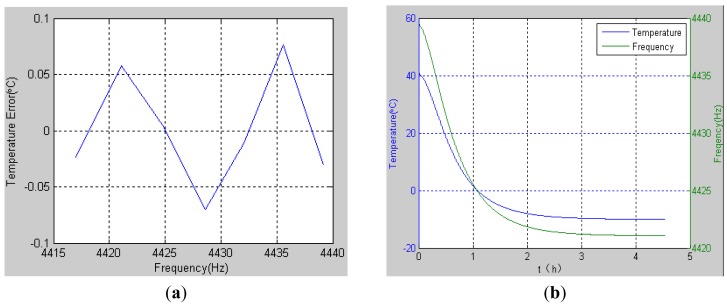
Temperature calculated from the frequency. (**a**) Calculated error (**b**) Frequency and temperature curves.

Using Equation (18), the temperature of resonator can be inversely calculated from the frequency which is the direct output from the HRG primary control loop in digital form. The calculation error is listed in Table 2 and the error is also shown in Figure 5(a). Additionally, the temperature calculated from the frequency is shown in Figure 5(b), which corresponds to the temperature change in Figure 3(a).

The largest deviation of the temperature error was below 0.1 °C in a temperature interval from −20 °C to 40 °C, which is close to the actual temperature. The experimental results indicate that the variation stability is very small, with a tolerance of less than 0.1 °C.

Based on the analysis above, it can be concluded that the natural frequency of the HRG is relative to the temperature and each temperature degree only corresponds to one natural frequency. Consequently, the natural frequency of the HRG, which can be easily obtained at any time, can be regarded as a high precision index of the temperature of the resonator. As a result, the frequency which is transmitted by the HRG system in real time can be used for HRG temperature compensation.

## 4. Temperature Model and Compensation of HRG Based on Its Natural Frequency

Based on the work above, we can compensate the output of HRG when the temperature is changing. Under FTR mode, the reference-phase loop and amplitude-control loop are employed to control the variation of the primary vibration pattern, which maintains the vibration at its natural frequency and at a constant amplitude [4]. Since the natural frequency signal is continuously given by the HRG output, on the basis of frequency changes and its change rates induced by the temperature variation, a compensation model for HRG is established, and then the compensations are realized by using a stepwise linear regression method.

### 4.1. Stepwise Linear Regression Method

Suppose y is an arbitrary variable, and its relation with the independent variables *x_i_,i* = 1,2…*n* is:

(19) $$y=b_{0}+b_{1}x_{1}+\ldots +b_{n}x_{n}+ɛ$$

where *b*_0_,*b*_1_,…,*b_n_* are the regression coefficients, and *ε* is an arbitrary error. Regression analysis is to evaluate the value of *b*_0_,*b*_1_,…,*b_n_* by using the independent variables *x_i_,i* = 1,2…*n* and the dependent variable y. The Equation (19) can be rewritten as:

(20) Y=Xb+ɛ

In Equation (20)*Y* is a group of observed data; *X* is a matrix of known independent variable; ***b*** is an unknown vector; *ε* is an error vector, and *E*(*ε*) = 0, *D*(*ε*) = *σ*^2^*I_n_*. The Equation (20) is called as a linear regression model, which uses the least-squares method to find the best coefficient vector ***b***, making the sum of the error square to the least. That is:

$$J=\left\|Y-X\hat{b}\right\|=\min{\left\|Y-Xb\right\|}^{2}$$

As a result, ***b̂*** is the least square estimate of ***b***. The necessary condition for the least error is:

$$\frac{\partial J}{\partial \hat{b}}=\frac{\partial }{\partial \hat{b}}{\left(Y-X\hat{b}\right)}^{T}(Y-X\hat{b})=-2X^{T}Y+2X^{T}X\hat{b}=0$$

and then:

$$\hat{b}={\left(X^{T}X\right)}^{-1}X^{T}Y$$

The sufficient condition for the least error is:

$$\frac{\partial }{\partial \hat{b}}(\frac{\partial J}{\partial \hat{b}})>0$$

As a systematic method, stepwise regression involves adding or removing terms from a model on the basis of their statistical status in a regression. If a term is not currently in the model, the null hypothesis is that the term would have a zero coefficient if added to the model. If there is sufficient evidence to reject this null hypothesis, the term will be then added to the model. Conversely, if a term is currently in the model, the null hypothesis is that the term has a zero coefficient, if evidence is not enough to reject the null hypothesis, the term will be removed from the model. To be brief, stepwise regression is widely used since it is unbiased and has a minimum variance among all unbiased estimators formed from linear combinations of the response data by the Gauss-Markov theorem.

### 4.2. Temperature Compensations Realized by Stepwise Regression Method

In the real work conditions for the HRG, the frequency change rates are different from each other because the heat field is uncertain and the heat conduction is uneven. Therefore, a drift model based on the frequency changes and frequency change rates is applied to make temperature compensations for the HRG. Besides, considering the coupled terms of frequency changes and the frequency change rates, three order temperature model can be established:

(21) $$B=B_{0}+\sum_{i=1}^{3}k_{i}f^{i}+k_{4}\frac{df}{dt}+k_{5}{\left(\frac{df}{dt}\right)}^{2}+k_{6}{\left(\frac{df}{dt}\right)}^{3}+k_{7}f\frac{df}{dt}+k_{8}f^{2}\frac{df}{dt}+k_{9}f{\left(\frac{df}{dt}\right)}^{2}$$

where *f* stands for the frequency change; B stands for the output bias and *k_i_* stands for the coefficients about frequency and frequency change rates. The compensation model above is composed of nine unknown variables, and finally generates a compensation model by stepwise linear regression, to which the significant items are added and from which the insignificant items are removed. At last, a frequency-bias model is shown as follows:

(22) $$B=B_{0}+\sum_{i=1}^{3}k_{i}f^{i}+k_{4}\frac{df}{dt}+k_{5}{\left(\frac{df}{dt}\right)}^{2}+k_{6}{\left(\frac{df}{dt}\right)}^{3}+k_{7}f\frac{df}{dt}$$

where a *B*_0_ represents the constant bias and it has no relation to the temperature; *k*_1_ − *k*_3_ represent the frequency change item coefficients, showing the change trend of the bias related to the frequency(temperature). *k*_4_ − *k*_5_ are the coefficients of frequency change rates; *k*_7_ is the coefficient of frequency change coupled with frequency change rates, showing the effect on the bias of HRG by their combination.

**Figure 6.**
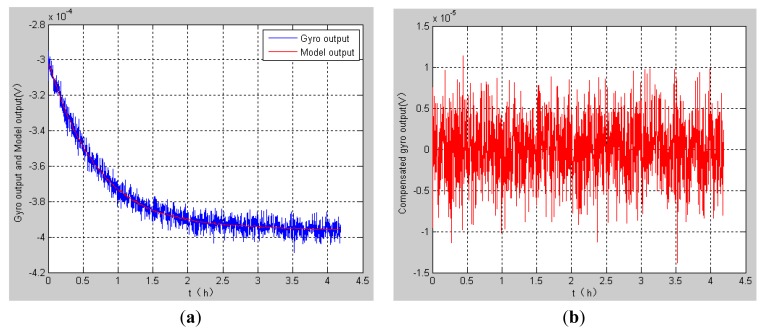
The output of the gyro. (**a**) The uncompensated output of the gyro from room temperature to −20 °C; (**b**) The compensated output of the gyro.

We make compensations for the drift of HRG by using the model described above. The blue curve in Figure 6(a) shows the output of the HRG from room temperature to −20 °C. As the temperature decreases, the gyroscope output also decreases as shown in Figure 6(a). Then the bias stability can be calculated from the original data, which is 3.0143 °/h. The red curve in Figure 6(a) is the compensation model output, from which the gyroscope output can be compensated and the compensated output is shown in Figure 6(b). The bias stability calculated from the compensated data is 0.5848 °/h, which basically reaches the constant temperature precision. In a word, the compensation effect is very obvious.

## 5. Conclusions

The external temperature changes have a strong effect on the HRG, for example, the material properties such as Young's modulus, the radius of the resonator and so on change because of the heat; the excite electrodes, resonator and pick-off electrodes displace irregularly due to the heat deformation, and all those phenomena result in bias drift decreasing the degree of precision of the HRG. As a result, it is of vital necessity to observe the influence on the HRG output by the temperature changes and then compensate for it. Only in this way, can the performance of the HRG be improved. In this paper, the relationship between temperature and frequency are firstly established, and then we compensate for the output of HRG by the frequency changes through its relation to the temperature changes. This method reduces the complexity of the compensation without using a temperature sensor. More importantly, it can be found that the experiments give a satisfactory result by using this compensation method, and it significantly improves the temperature stability of the HRG.

## Figures and Tables

**Table 1.** Different natural frequencies of HRG in correspondence with different temperatures.

**Temperature (°C)**	**Natural Frequency (Hz)**
−20	4,416.97
−10	4,421.05
0	4,424.90
10	4,428.56
20	4,432.07
30	4,435.56
40	4,439.20

**Table 2.** Temperature calculated from the frequency and the calculation errors.

**Frequency(Hz)**	**4,416.97**	**4,421.05**	**4,424.90**	**4,428.56**	**4,432.07**	**4,435.56**	**4,439.20**
Actual temperature(°C)	−20	−10	0	10	20	30	40
Calculate temperature (°C)	−19.976	−10.058	−0.0026	10.070	20.011	29.924	40.031
Calculated error(°C)	−0.024	0.0578	0.0026	−0.070	−0.011	0.076	−0.031
